# Machine diagnosis of chronic obstructive pulmonary disease using a novel fast-response capnometer

**DOI:** 10.1186/s12931-023-02460-z

**Published:** 2023-06-02

**Authors:** Leeran Talker, Daniel Neville, Laura Wiffen, Ahmed B. Selim, Matthew Haines, Julian C. Carter, Henry Broomfield, Rui Hen Lim, Gabriel Lambert, BRS Study Team, Jonathon Winter, Andrew Gribbin, Milan Chauhan, Ruth De Vos, Paul Kalra, Selina Begum, Barbara Robinson, Bernadette Mundy, Heather Rutter, Karen Madronal, Scott T. Weiss, Gail Hayward, Thomas Brown, Anoop Chauhan, Ameera X. Patel

**Affiliations:** 1 TidalSense Limited, 15a Vinery Rd, Cambridge, CB1 3DN UK; 2 Portsmouth Hospitals University NHS Trust, Portsmouth, UK; 3 Nuffield Department of Primary Care Health Sciences, NIHR Community Healthcare MedTech and IVD Cooperative, University of Oxford, Oxford, UK; 4 Channing Division of Network Medicine, Department of Medicine, Harvard Medical School, Boston, MA USA

**Keywords:** Chronic Obstructive Pulmonary Disease, Machine learning, Diagnosis

## Abstract

**Background:**

Although currently most widely used in mechanical ventilation and cardiopulmonary resuscitation, features of the carbon dioxide (CO_2_) waveform produced through capnometry have been shown to correlate with V/Q mismatch, dead space volume, type of breathing pattern, and small airway obstruction. This study applied feature engineering and machine learning techniques to capnography data collected by the N-Tidal™ device across four clinical studies to build a classifier that could distinguish CO_2_ recordings (capnograms) of patients with COPD from those without COPD.

**Methods:**

Capnography data from four longitudinal observational studies (CBRS, GBRS, CBRS2 and ABRS) was analysed from 295 patients, generating a total of 88,186 capnograms. CO_2_ sensor data was processed using TidalSense’s regulated cloud platform, performing real-time geometric analysis on CO_2_ waveforms to generate 82 physiologic features per capnogram. These features were used to train machine learning classifiers to discriminate COPD from ‘non-COPD’ (a group that included healthy participants and those with other cardiorespiratory conditions); model performance was validated on independent test sets.

**Results:**

The best machine learning model (XGBoost) performance provided a class-balanced AUROC of 0.985 ± 0.013, positive predictive value (PPV) of 0.914 ± 0.039 and sensitivity of 0.915 ± 0.066 for a diagnosis of COPD. The waveform features that are most important for driving classification are related to the alpha angle and expiratory plateau regions. These features correlated with spirometry readings, supporting their proposed properties as markers of COPD.

**Conclusion:**

The N-Tidal™ device can be used to accurately diagnose COPD in near-real-time, lending support to future use in a clinical setting.

*Trial registration:* Please see NCT03615365, NCT02814253, NCT04504838 and NCT03356288.

**Supplementary Information:**

The online version contains supplementary material available at 10.1186/s12931-023-02460-z.

## Background

Chronic Obstructive Pulmonary Disease (COPD) is a progressive respiratory disease most associated with a smoking history. It is the third most common cause of mortality worldwide [1], causing 3.28 million deaths in 2019 and affecting 212 million people globally [2].

Although no cure currently exists, early diagnosis and treatment are important to improve lung function and quality of life and reduce exacerbations [3]. The clinical standard for COPD diagnosis is spirometry, which relies upon a patient’s ability to exhale forcefully. However, a major disadvantage of spirometry is that it is effort-dependent and thus unreliable and non-specific [4]. In addition, it is effective at detecting latter stages of disease but has lacked reliability in screening for early and asymptomatic cases [5]. This limitation poses challenges in diagnosing a slowly progressing disease such as COPD with a long asymptomatic pre-prodromal phase [6]. It has been estimated that in the UK, only between 9.4 and 22% of those with COPD have been diagnosed [7], in part due to spirometry’s poor precision of only 63% [8].

Capnography is a widely used technique in critical care and anaesthetics. It has been suggested that features of a high-resolution capnogram could be used to identify physiologic patterns associated with respiratory diseases such as COPD [9]. TidalSense’s N-Tidal™ device (MHRA reference: 5182) has made it possible to measure CO_2_ concentration reliably and accurately via tidal breathing through a mouthpiece, with a greater time resolution than previously possible [10], making the technique an appealing alternative to spirometry.

The objective of this paper was to apply machine learning (ML) techniques to capnography data collected using the N-Tidal™ device across four clinical studies. The aim was to build a classifier that could distinguish capnograms of patients with COPD from those without COPD using only one breath recording, while maintaining explainability of outputs, with a clear reference to respiratory physiology. This data was analysed to assess the capability of N-Tidal™ to function as a point-of-care diagnostic tool for COPD.

## Methods

### Participants

It was vital to ensure any diagnostic classifier could distinguish COPD from other conditions that could cause a similar symptom burden at presentation, since many patients undergoing physiological testing for COPD may have an alternative diagnosis. Therefore, the capnograms used in this analysis were collected from four different observational cohort studies, known as GBRS, ABRS, CBRS and CBRS2, that together provided a dataset with appropriate heterogeneous medical conditions. A summary of these studies with their objectives can be found in Additional file 1. They included patients with COPD, asthma, heart failure, pneumonia, breathing pattern disorder, motor neuron disease, sleep apnoea, bronchiectasis, pulmonary fibrosis, tracheobronchomalacia, as well as healthy participants.

In patients with COPD, diagnoses were made according to NICE guidelines with most patients being Global Initiative for Chronic Obstructive Lung Disease (GOLD) stages three or four (representing severe and very severe disease respectively). Diagnostic criteria used for other conditions, including asthma, are in Additional file 1 alongside each protocol’s inclusion and exclusion criteria. In addition to the four studies noted above, capnography data was collected from 34 volunteers without any respiratory disease between December 2015 and January 2022. Though not part of a formal study, these volunteers provided written informed consent and were screened by a medical doctor to ensure they did not have any confounding cardiorespiratory disease or other co-morbidities. All subjects across the four studies gave informed consent, and their data was handled according to all applicable data protection legislations, including the EU/UK General Data Protection Regulation.

Ethical approval was obtained from the South Central - Berkshire - Research Ethics Committee (REC) for GBRS and ABRS, the Yorkshire and the Humber REC for CBRS and the West Midlands Solihull REC for CBRS2.

### Procedures

In all studies, capnography data was serially collected using the N-Tidal™ device twice daily for varying lengths of time according to each study’s protocol. The N-Tidal™ device is a CE-marked medical device regulated in the UK and EU, and has been designed to take accurate, reliable recordings of respired partial pressure of CO_2_ (pCO_2_) directly from the mouth. The N-Tidal™ device is unique in its ability to accurately measure pCO_2_ from high CO_2_ to background levels, with a fast response time, meaning that quick changes in the geometry of the pCO_2_ waveform are captured.

Patients performed normal tidal breathing through the N-Tidal™ device for 75 s through a mouthpiece in an effort-independent process. This duration was selected as it ensured adequate data collection (assuming that some breaths were likely to be noisy and may require elimination prior to analysis), while also ensuring that data collection was relatively short for patients. CO_2_ was sampled at 10 kHz and reported at 50 Hz providing a level of resolution not possible with alternative capnometers [10]. Reporting at 50 Hz was done to improve signal-to-noise ratio, and to ensure CO_2_ data could be quickly transmitted over mobile networks while still ensuring high temporal resolution. A single episode of use (breath recording) produced a single capnogram, with each respiratory cycle (inspiration and expiration) forming a single waveform.

In addition to capnometry data, the following data was also collected for all four studies: basic demographics, spirometry (taken on the same day as N-Tidal™ data), and medical history. Other clinical and questionnaire data varied across studies (see objectives and inclusion/exclusion criteria in Additional file 1).

### Feature engineering

**Fig. 1:**
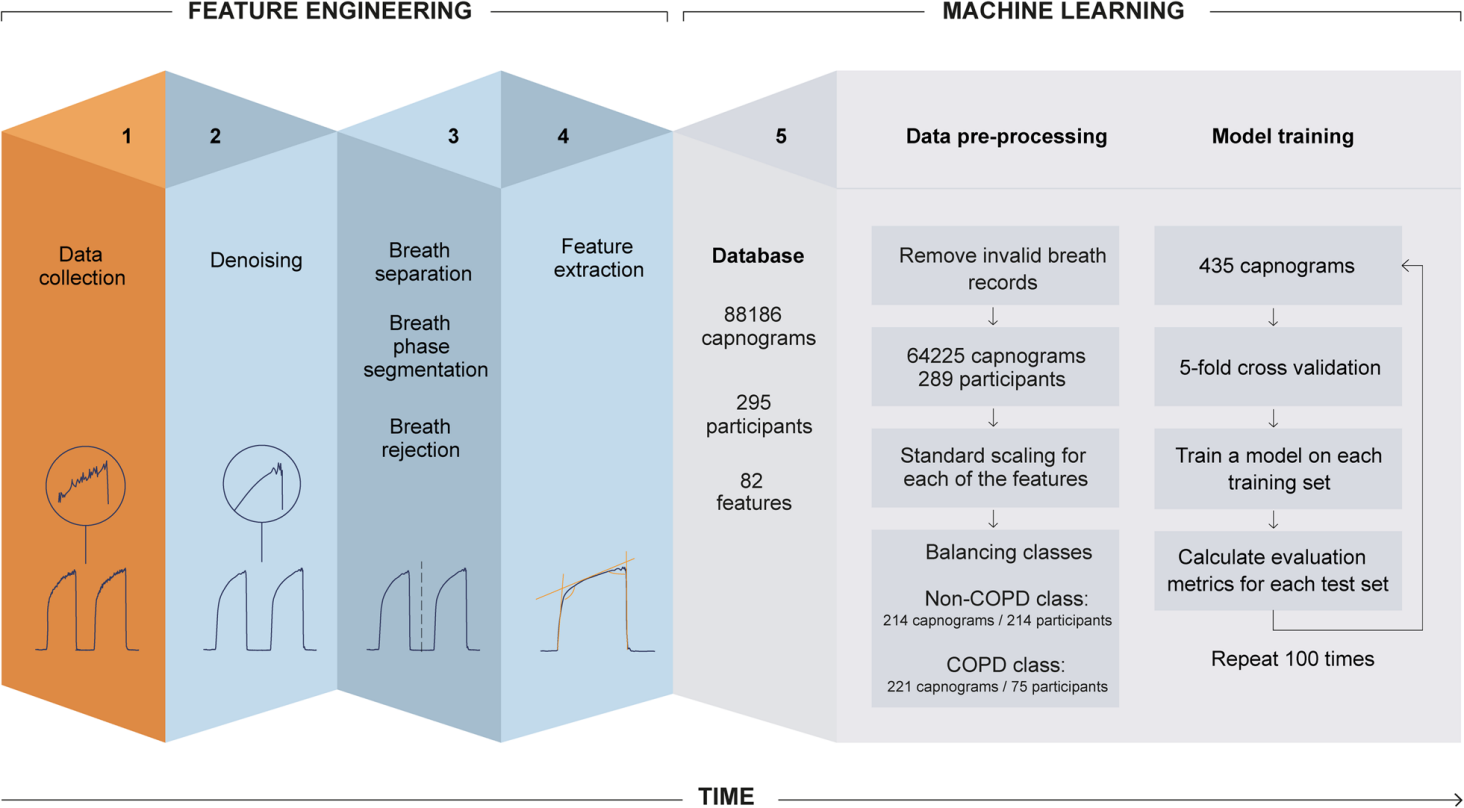
High-level overview of the feature engineering and machine learning pipeline used for data processing, feature engineering and model training of fast-response CO_2_ data collected through the N-Tidal™ device

For each capnogram collected using a N-Tidal™ device, raw pCO2 data was first denoised (see Additional file 1 for waveform correlations before and after denoising), then individual breaths were separated and breath phases segmented before being stored in a secure cloud database (Fig. 1). Individual anomalous breaths within a capnogram that could not be processed were identified and excluded from analysis using automated software built into the N-Tidal™ cloud platform. Reasons for exclusion included: breathing through the nose, water vapour condensation-compromised sensor readings, incomplete breaths, noisy breaths caused by swallowing or coughing into the device, or cardiogenic oscillations. Each of these artefacts creates specific patterns of discontinuity in the CO_2_ waveform which alters the geometry of the waveform (e.g. swallowing, coughing artefacts and cardiogenic oscillations), or substantially impacts the signal-to-noise ratio (e.g. nose breathing or condensation-compromise artefacts). In both cases, these breaths were automatically flagged for exclusion by the N-Tidal™ cloud platform and were not included in further analysis.

To generate features for the ML classification task, two categories of information were captured: geometric characteristics of the waveform associated with each breath (referred to as ‘per breath features’); and features of the whole capnogram, such as respiratory rate or maximum end-tidal CO_2_ (ETCO_2_), referred to as ‘whole capnogram features.’

**Fig. 2:**
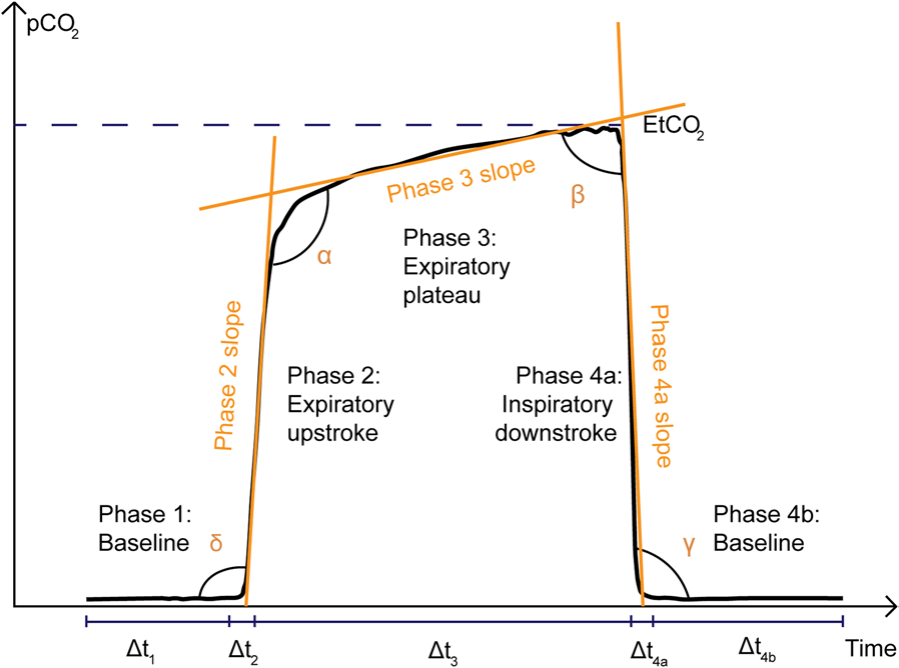
Illustration of a capnogram waveform and its phases and angles. Phase 1 is the inspiratory baseline, Phase 2 is the expiratory upstroke (representing the first phase of exhalation), Phase 3 is the expiratory plateau (representing the end of exhalation), Phase 4a is the inspiratory downstroke (representing the first phase of inhalation), and Phase 4b is the inspiratory baseline. Note that the start of Phase 1 and the end of Phase 4b may technically be considered part of the same phase

For each capnogram, 77 per-breath features and 5 whole capnogram features were derived. As the number of breaths per capnogram varied, the median and standard deviation were calculated for the per-breath features in each capnogram. These features included the following: α, β, γ, and δ angles (Fig. 2) [11–13]; gradients and residuals derived from fitting curves to phases, such as the expiratory plateau [14]; absolute and short-term variability of pCO_2_ [15]; curvature and other higher-order time-based features such as the ratio of the expiratory to inspiratory phase [12, 13, 16]; and area under the curve (AUC), which is commonly calculated in volumetric capnography [16, 17]. These features have a basis in the respiratory physiology literature, and in many cases have been hypothesized to relate to clinical airway obstruction.

Any breaths where the full feature set could not be calculated were also automatically excluded from analysis, and further checks were carried out manually to ensure that all condensation-compromised breaths had been excluded by the automated methods.

### Machine learning

Following pre-processing and feature engineering, each feature was normalized and scaled to a mean of zero and standard deviation of 1. Significant class imbalances are known to bias machine learning (ML) models towards low predictive accuracy on an under-represented target [18]. To address this, the first capnogram from each participant in the majority class (non-COPD) was retained, and the first three capnograms from each participant in the minority class (COPD) were retained. This approach represented a small fraction of the total breath records available, but using a small number of capnograms per patient had several advantages. First, it ensured that the models would be less likely to overfit on features of individual patients, such as confounding co-morbidities, enabling them to learn the general capnographic characteristics of COPD across a larger population. Secondly, it mimicked the anticipated real-world application of the N-Tidal™ device and ML model, namely, to diagnose COPD based on a single breath recording.

Next, 20% of the dataset was put aside as an unseen test set and the remaining 80% was used for model training and testing. During training, a group-stratified five-fold cross-validation procedure was applied, which involved dividing the dataset into five folds, ensuring no patient overlap between test sets and training sets. For each iteration, data was sampled from four folds for training, with the remaining fold left out for testing. For example, one iteration of training used 348 capnograms from 231 patients in the training set, and 87 capnograms from 58 patients in the test set. This ensured that the data used to test performance of the model was not included in the training process. The mean performance of all five iterations produced the results of the overall model performance. In addition, the performance variability across all five folds gave a measure of model generalizability. Upon completion of the group-stratified five-fold cross-validation procedure, the best model was then tested on the unseen test set to verify robustness of model performance in a completely unseen set, which was designed to be indicative of real-world performance.The capnogram features were fed to three different ML models: logistic regression (LR); extreme gradient boosted trees (XGBoost); and a support vector machine (SVM) with a linear kernel. These models were chosen due to their more interpretable nature. Deep learning methods were omitted to maintain explainability and to minimize model complexity. The five-fold cross-validation, training, and testing steps were repeated 100 times to calculate the average of the reported metrics. Finally, the capnograms where the model was less confident in assigning to a class (COPD vs. non-COPD) and those that were misclassified were investigated.

### Statistical methods

The Python scikit-learn package was used to produce each of the following: sensitivity; specificity; negative predictive value (NPV); positive predictive value (PPV); receiver operator characteristic (ROC) curves; micro-averaged area under ROC (AUROC); and confusion matrix (for a decision boundary of 0.5). The most significant features driving model learning were extracted to understand which features of the capnogram waveform were most predictive of COPD. To investigate the relationship between traditional diagnostic methods and these most predictive features, the median of each feature, for each patient, was plotted against the patient’s paired spirometry result, and correlations were calculated.

### Data availability

The datasets analysed in this publication are not publicly available for data protection reasons.

## Results

Between 6 December 2015 and 31 January 2022, 88,186 capnograms were collected from 295 patients. On average, each patient collected 299 capnograms over 179 days. Demographic data was collated (Table 1).

**Table 1:** Demographic information from the four studies and the separate healthy volunteer cohort

	COPD (N = 80)	Non-COPD (N = 215)	Overall (N = 295)
Age	67 (61–73)	52 (39–64)	58 (45–67)
Gender (female)	39 (48.8%)	133 (61.9%)	176 (58.5%)
BMI (kg/m^2^)	25.2 (22.1–30.0)	28.9 (24.3–34.3)	27.9 (23.4–33.3)
Smoking history			
Current smoker	10 (12.0%)	9 (4.2%)	19 (6.4%)
Ex-smoker	66 (82.0%)	71 (36.8%)	137 (50.7%)
Never smoked	0 (0%)	113 (58.5%)	113 (41.9%)
Pack years (all)	40.0 (31.2–58.8)	0.0 (0.0–4.0)	1.0 (0.0–20.0)
Current smoker	47.7 (34.5–57.5)	23.3 (15.0–33.8)	31.0 (16.5–50.0)
Ex-smoker	40.0 (31.5–58.3)	5.0 (2.0–10.0)	12.5 (3.8–38.0)

Categorical data is given as a number with its percentage of the total (n (%)). Continuous data is given as (median (Q1–Q3)). Smoking history was absent for 31 participants and pack years was absent for 58 participants

### Classification performance

**Fig. 3:**
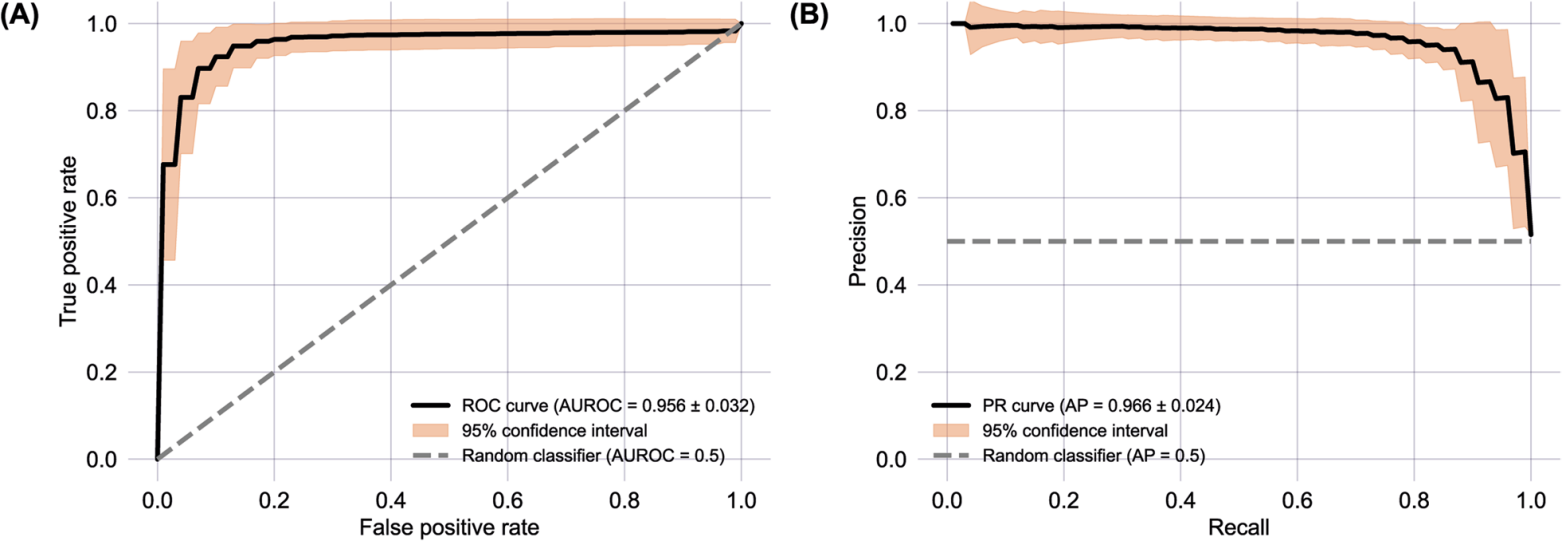
**A** Receiver operator characteristic (ROC) curve for the LR model, reported with results of a theoretical ‘random’ classifier with no predictive power, and a 95% confidence interval, calculated across all 100 iterations. **B** Precision-Recall Curve for the LR model, reported with the results of a theoretical ‘random classifier’ and the average precision (AP)

In classifying COPD vs non-COPD participants, XGBoost showed the best performance on the test set (P < 0.05, see Additional file 1) with AUROC, specificity, PPV, accuracy, and NPV at 0.985, 0.914, 0.914, 0.913 and 0.912 respectively (Table 2). Model performance was highly consistent between iterations as shown by low standard deviation (Table 2). As performance for all models was similarly high, further analysis is only presented for the LR model, as it is the most explainable. These metrics and those in Fig. 3 (including standard deviations across folds) are an average across the 100 iterations. In other words, five-fold cross validation was performed 100 times, and the results were averaged across all iterations.

**Table 2:** Machine learning model performance on the leave out test set during the cross-validation procedure, averaged across 100 iterations (shown as mean across iterations ± standard deviation), for each of the three models built: logistic regression (LR), extreme gradient boosted trees (XGBoost), and support vector machine (SVM) with a linear kernel

	Accuracy	AUROC	Sensitivity	Specificity	Negative predictive value (NPV)	Positive predictive value (PPV)	Classifier build time (seconds)
LR	0.894 ± 0.041	0.956 ± 0.032	0.875 ± 0.073	**0.914 ± 0.044**	0.876 ± 0.067	0.914 ± 0.042	0.027 ± 0.009
XGBoost	**0.913 ± 0.035**	**0.985 ± 0.013**	**0.915 ± 0.066**	0.914 ± 0.045	**0.912 ± 0.063**	**0.914 ± 0.039**	0.600 ± 0.082
SVM	0.906 ± 0.036	0.975 ± 0.019	0.900 ± 0.067	0.911 ± 0	0.898 ± 0.064	0.914 ± 0.064	**0.012 ± 0.0003**

The highest performance (across all models), for each of the metrics reported is highlighted in bold

Table 3 shows the performance on the unseen test set. SVM was the top performing classifier across all metrics with accuracy, sensitivity and specificity at 0.933, 0.956 and 0.911. Differences in performance between the LR and XGBoost model were marginal. LR performed slightly better than XGBoost with an accuracy, sensitivity and specificity of 0.911, 0.956 and 0.867 respectively.

**Table 3:** Machine learning model performance on the unseen test set for each of the three models built: logistic regression (LR), extreme gradient boosted trees (XGBoost), and support vector machine (SVM) with a linear kernel

	Accuracy	Sensitivity	Specificity	Negative predictive value (NPV)	Positive predictive value (PPV)
LR	0.911	0.956	0.867	0.951	0.878
XGBoost	0.900	0.956	0.844	0.950	0.860
SVM	**0.933**	0.956	**0.911**	**0.953**	**0.915**

The highest performance (across all models), for each of the metrics reported is highlighted in bold

### Post-hoc analysis

**Fig. 4:**
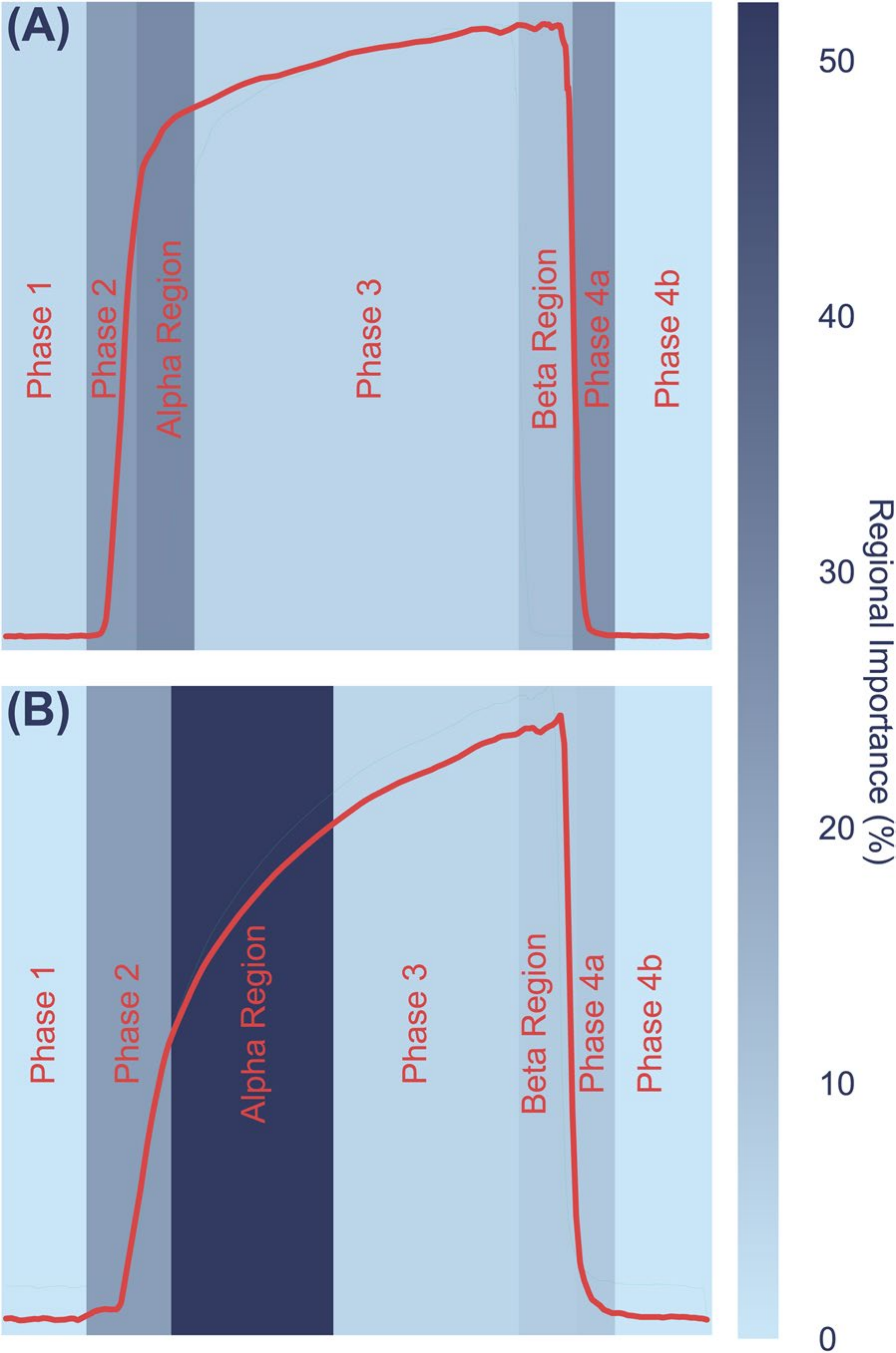
Average weighted feature importance by capnogram waveform region, where weighted features were calculated as the magnitude of the product of the normalized feature value and the feature importance. **A** shows an example for a non-COPD waveform, and **B** shows an example for a COPD waveform

**Fig. 5:**
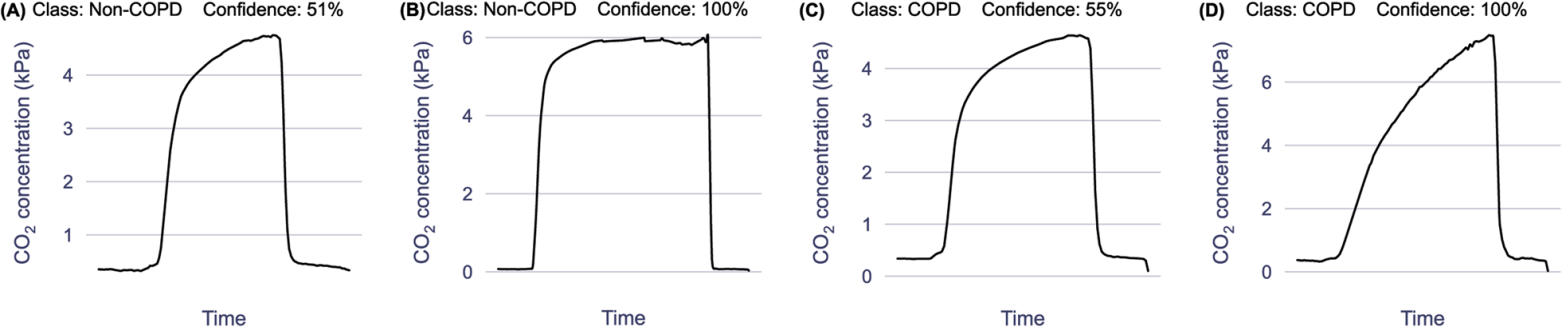
Average waveforms (of correctly classified capnograms), with prediction confidence expressed as a % (averaged across 100 iterations). **A** and **B** are two examples of the non-COPD class with the corresponding confidences and **C** and **D** show two examples of the COPD class with the corresponding confidences. **A** and **C** are examples with low prediction confidence and **B** and **D** are examples with high prediction confidence

**Fig. 6:**
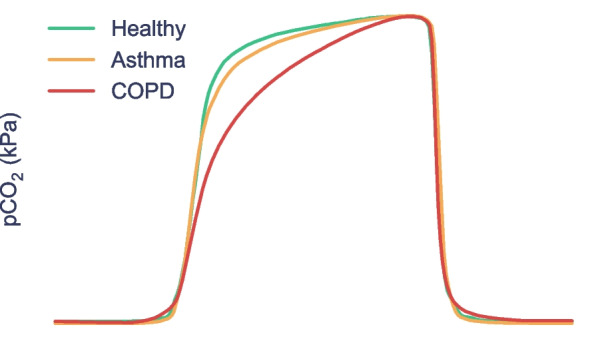
Average capnogram waveforms across all healthy, COPD and asthmatic patients. As seen from the example waveforms, clear differences in capnogram geometry, specifically the alpha region, exist between all three waveforms with the COPD waveform diverging markedly from the healthy and asthmatic waveforms

A post-hoc evaluation of the predictive model was conducted to identify which capnogram features best distinguished patients with and without COPD. The relative feature importances for driving learning in the LR model were determined and the region of the capnogram waveform from which the features were extracted was used to construct the importance map for non-COPD and COPD waveforms in Fig. 4. The heatmap value of each region represents an average of the weighted feature importance for that region, across all features assigned to that region. The weighted feature importance is calculated as the normalized value for that feature multiplied by the feature importance. Features associated with phase 2 and phase 3 of the capnogram waveform (the exhalation phase) were found to be the most important drivers of learning. Figure 5 shows the average waveform of four correctly classified capnograms: two each for low and high model confidence, where confidences were calculated as the mean across 100 iterations. In addition, Fig. 6 shows the average waveform across a 75 s capnogram for all healthy volunteers, asthmatic patients and COPD patients. Differences in the shape of the alpha angle region can be observed across the three groups, with COPD patients showing the most marked divergence in geometry from the healthy waveform.

**Fig. 7:**
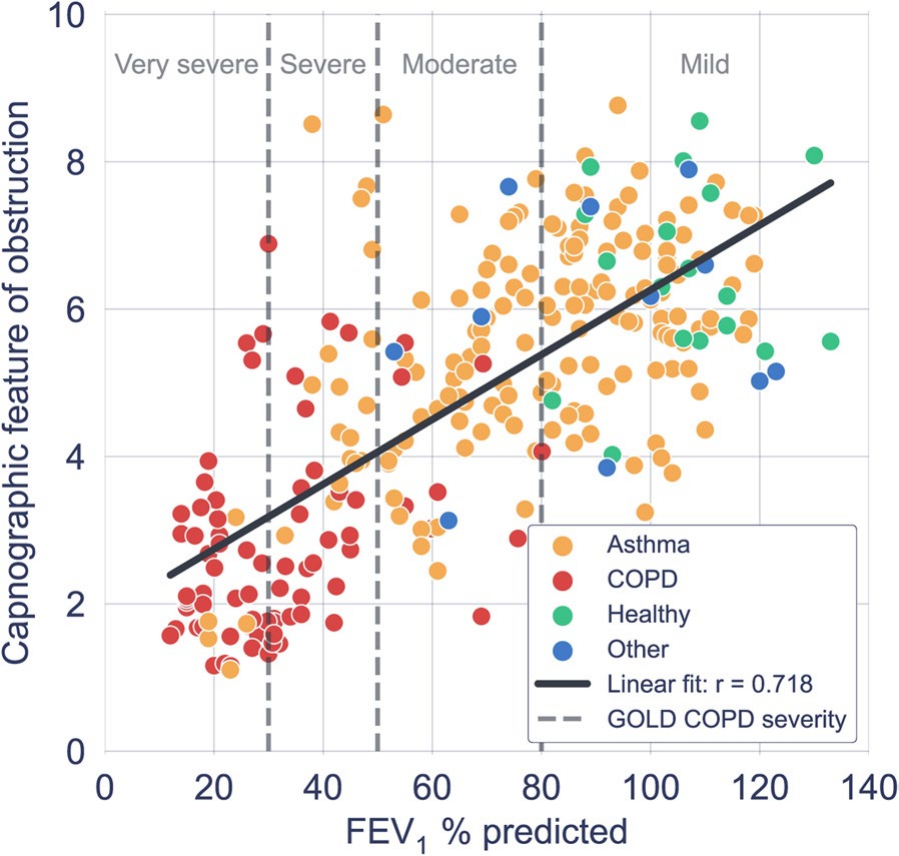
Scatter plot of an example of one of the most important capnography features driving learning in the logistic regression model, versus % predicted FEV1 from paired spirometry data. Each point represents a single paired capnogram. The correlation coefficient was 0.718

To understand the relationship between the capnography features driving the learning and spirometry metrics typically reported in COPD, the  % predicted FEV1 for each participant was correlated with the corresponding 20 most important features as determined by the LR model, using a Spearman’s rank correlation coefficient, ρ (since not all relationships were linear). This was only calculated for patients with paired spirometry (177 patients). Of the 20 features, six showed significant correlations |ρ|> 0.5. An example of one of the most important feature’s relationship  with % predicted FEV1 can be found in Fig. 7, where a linear relationship prompted the calculation of the product moment correlation coefficient (r) which was 0.718.

### Model bias and model performance across repeat readings

Next, to assess whether there was significant variation in classification results between repeat capnography readings taken over time, the classification accuracy and the standard deviation of the LR model’s output probabilities was calculated for all of the capnograms of each patient in the test fold (as opposed to evaluating only one or three capnogram(s) per patient). The results were averaged over 100 iterations to ensure consistency of the result, and the results reported in Table 4 are mean values calculated over all the patients in each disease category for both metrics.

**Table 4:** Classification accuracy and standard deviation of model confidence during the cross-validation procedure, by disease group, for all capnography data collected by each participant in the test set, averaged over disease group and again over all 100 iterations

	Number of patients	Average number of capnograms per patient	Classification accuracy per patient	Std. of probability of COPD per patient
Healthy	34	242	0.968 ± 0.006	0.072 ± 0.007
Asthma	141	289	0.923 ± 0.005	0.073 ± 0.002
COPD	75	150	0.891 ± 0.009	0.101 ± 0.004
Heart failure	10	169	0.798 ± 0.025	0.142 ± 0.011
Breathing pattern disorder	10	124	0.981 ± 0.003	0.086 ± 0.006
Pneumonia	16	46	0.839 ± 0.016	0.105 ± 0.007
Motor neuron disease	3	95	0.800 ± 0.047	0.129 ± 0.016

An important aspect of model validation was to investigate demographic bias. For this, patients that were misclassified more than 50% of the time across all 100 iterations were termed ‘misclassified’. These misclassification rates were stratified by birth sex and COPD status, highlighting that discrepancies were small and misclassification was not overly skewed towards a single characteristic (Table 5). Additionally, the difference between the medians of age distributions of the misclassified and correctly classified capnograms was not statistically significant (Mann–Whitney U = 8000, P = 0.92 two-tailed), indicating that age did not bias the chances of misclassification. The same procedure was performed for BMI, yielding similar outcomes (Mann–Whitney U = 7700, P = 0.57, two-tailed).

**Table 5:** Misclassification rates of each sex versus disease group for the logistic regression (LR) model

	Female	Male	Total
Non-COPD	7.6% (10/132)	11.0% (9/82)	8.9% (19/214)
COPD	8.3% (3/36)	7.7% (3/39)	8.0% (6/75)
Total	7.7% (13/168)	9.9% (12/121)	8.7% (25/289)

## Discussion

### Robustness of prediction accuracy between models, test sets and repeat measurements over time

The aim of this study was to demonstrate that a single breath recording from the N-Tidal™ fast-response capnometer, in conjunction with a machine-learning-derived diagnostic classifier, could be used to accurately classify those patients with and without COPD. It was found that the performance of the best machine learning model (XGBoost), when tested during cross-validation on the test set (P < 0.05, see Additional file 1), provided a class balanced AUROC of 0.985 ± 0.013 and positive predictive value (PPV) of 0.914 ± 0.039. The performances of the three models (LR, XGBoost and SVM with a linear kernel) were very similar. Statistical testing carried out between iterations demonstrates high levels of consistency (Table 2) and therefore high model stability. For instance, the largest difference in sensitivity between models was 0.04 and the largest difference in positive predictive value between models was 0.001. When tested on the unseen test set (Table 3), performance was similar to that on the cross-validation test set with marginal observed differences. The similarity in classification performance across all three models, and consistent high classification accuracy (Table 2, Additional file 1) provides confidence in the thesis that COPD can be reliably detected from geometric properties of the CO_2_ waveform. In addition, the fact that similarly high model performance on the unseen test set was observed indicates that the models were not overfitting to the training or test sets during five-fold cross-validation, as the unseen test set used data from completely separate (unseen) patients. This provides confidence in model performance when deployed in the real world, provided that our training population is representative of the population at large.

Furthermore, the prediction accuracy by disease state implies that healthy individuals and patients with asthma or COPD are the easiest to classify (healthy being the easiest), with 0.968 ± 0.006, 0.923 ± 0.005 and 0.891 ± 0.009 classification accuracies respectively (Table 4) across repeat readings. This experiment evaluates how consistently the model classifies each patient with the same diagnosis. The more consistent classification results for healthy individuals may reflect a reduced variability in CO_2_ waveform shape over time, compared to individuals with an obstructive airway disease with an element of airway variability. Interestingly, nearly all patients with asthma, and many patients with COPD, showed variability in waveform geometry over time, which may be a diagnostic indicator in its own right, and may represent a challenge with diagnosing patients from a single point in time recording.

### Interpretability and exploring the waveform features driving learning

The motivation for using XGBoost, LR and SVM with a linear kernel was to achieve a high level of interpretability, and ease of traceability to the individual features that most informed the model’s decision. This is not only important in providing clinicians with confidence in what waveform features are driving the model’s prediction, but also in providing reassurance that what the models are learning is in fact intrinsically tied to respiratory physiology which can be explained from a hypothesis-driven point of view. As a result, interpretability is now becoming an increasingly common requirement in guidelines for the implementation of machine learning in medical devices. The identified features (Fig. 4) contributing most to the model’s decision were from the α angle region and expiratory plateau, where the latter features quantified the concavity, height, and central timestamp of the expiratory plateau. Together, these features characterize the rate at which gas from the upper airways (poor in CO_2_) gives way to mixed alveolar gas from the lower airways (richer in CO_2_). A larger α angle, and a smaller value of these plateau features corresponds to greater concavity and greater airway resistance, likely due to an obstructed bronchospastic airway. The analysis supports the physiological plausibility of these features in underpinning the obstructive airway pathology of COPD, as a number of the most important features showed strong correlation to paired % predicted FEV1 data (Fig. 7). The average waveform, across the entire dataset, for healthy volunteers, asthma patients and COPD patients can be found in Fig. 6. We observed that COPD specifically, was associated with a more curved ‘shark-fin’ type waveform, whereas patients with asthma had a more preserved transition between expiratory upstroke and plateau phases. However, there was heterogeneity among asthma patients with some patients showing a more shark-fin waveform but others (with well-controlled disease) looking more healthy. The shark-fin type waveform is known to arise from differences in time constants of gas movement from different alveoli to the sensor, as a result of differing compliance and resistance of alveoli. The changes in compliance and resistance arise as a result of the airway remodelling that occurs in COPD. This changes the rate of transition of gas from alveoli to anatomical dead space (i.e. the expiratory upstroke). Traditionally, based on the anaesthesia literature, the shark-fin waveform has been associated with obstructive airway diseases in general. However, we were able to resolve differences between asthma and COPD, and in particular, different severities of asthma. This might represent a difference in the populations studied, i.e. the study of more severe asthmatics with remodelled airways in the anaesthesia literature. However, our hypothesis for why N-Tidal™ is able to resolve the difference more clearly between different obstructive airway diseases is due to the high temporal resolution and accuracy of sampling, particularly in the fast transition phases (i.e. upstroke and downstroke phases). This means that the CO_2_ signal from the N-Tidal™ sensor is not aliased during fast waveform transition phases, meaning that more subtle differences in curvature (and therefore more subtle differences in V/Q mismatch or alveolar compliance and resistance) can be resolved. This allows N-Tidal™ to be more sensitive at discriminating between different cardiorespiratory conditions.

### Exploring misclassification and model bias

The average waveforms of a selection of capnograms for which the model had varying predictive confidence can be seen in Fig. 5. Capnograms for which the model had the highest confidence were at the extremities of a square-shaped healthy waveform with smaller α angle and smaller expiratory plateau tangent (relative to the horizontal), or a shark-fin-shaped COPD waveform with larger α angle and larger expiratory plateau tangent (relative to the horizontal). Reassuringly, we did not find any significant association between prediction accuracy and demographic features (including age, birth sex, and BMI) through non-parametric statistical testing, indicating that there is no systematic bias in model performance with respect to demographic data collected on the studies.

Several factors may have contributed to the erroneous classification of a small group of patients as having COPD when they had asthma or other respiratory conditions. First, smokers and ex-smokers with a significant pack year history were present in the non-COPD group, suggesting there may be some patients with undiagnosed COPD. Of the 25 patients misclassified by the LR model, 12 were either current smokers or ex-smokers, and this group had a mean smoking pack year history of 22. Secondly, the ABRS study highlighted a subgroup of asthma patients whose capnometry was ‘COPD-like’; some patients in this subgroup were suspected to have airway remodelling through many years of severe, poorly-controlled asthma, creating a COPD-like physiologic picture.

### Limitations and scope for future work

The work presented in this article has a number of limitations. First, simpler ML models were used in keeping with the National Health Service Artificial Intelligence recommendations regarding algorithmic explainability [19]. It is important to understand the link between the features driving the ML model and the pathophysiological process under investigation, but this precludes ‘deep learning’ methods that may have enhanced model performance. Secondly, COPD patients in the source dataset were predominantly GOLD stages 3 or 4 and managed in secondary care and, therefore, not representative of the general COPD population. Ideally, a diagnostic model would be based on a prospective analysis of those with a clinical suspicion of COPD divided into those who did and did not ultimately have the condition. Thirdly, ground-truth labels could only be obtained using current diagnostic pathways, known to have their shortcomings and inaccuracies. Therefore, a misclassification of a participant by one of the models presented in this article could, in some cases, be caused by mislabelling or misdiagnosis. Finally, the variability in pathologies in the non-COPD group was limited to the datasets collected on the studies, and may fail to account for the full spectrum of heterogeneity that would be encountered in a real-world clinical setting. Regardless, the proposed methods managed to distinguish on capnography alone (without supplementary data such as smoking history), between COPD and a range of differential diagnoses for patients likely to be referred for respiratory physiological testing (including healthy volunteers), demonstrating its potential clinical benefit.

In summary, we demonstrate that the N-Tidal™ fast-response capnometer and cloud analytics pipeline can perform real-time geometric waveform analysis and machine-learning-based classification to accurately diagnose COPD within minutes of breathing into the device. In contrast to commonly used ‘black box’ machine learning methodologies, a set of highly explainable methods were used that can provide traceability for machine diagnosis back to individual geometric features of the pCO_2_ waveform and their associated physiological properties suggestive of obstructive airways disease.

## Supplementary Information

**Additional file 1: Table S1.** Summary of the four clinical studies from which the paper has drawn its data. **Table S2.** Model performance compared using a paired t-test. Each model is benchmarked 100 times producing a series of the following metrics: specificity, sensitivity, F1 and AUROC. For example, model A produces 100 repeats of the AUROC metric, model B the same. Hypothesis testing is then carried out on these two series. **Table S3.** Classification accuracy and standard deviation of the model confidence for all capnography data collected by each participant in the test set, averaged over disease group and again over all 100 iterations. **Figure S1.** A point represents the correlation between each patient’s capnogram before and after the denoising procedure is applied.
